# Microfluidic Device for Microinjection of *Caenorhabditis elegans*

**DOI:** 10.3390/mi11030295

**Published:** 2020-03-11

**Authors:** Reza Ghaemi, Justin Tong, Bhagwati P. Gupta, P. Ravi Selvaganapathy

**Affiliations:** 1 Department of Mechanical Engineering, McMaster University, Hamilton, ON L8S 4L8, Canada; reza.rkh@gmail.com; 2 Department of Biology, McMaster University, Hamilton, ON L8S 4L8, Canada; homie_tong@hotmail.com (J.T.); guptab@mcmaster.ca (B.P.G.)

**Keywords:** *C. elegans*, microinjection, microfluidics, compliant mechanism

## Abstract

Microinjection is an established and reliable method to deliver transgenic constructs and other reagents to specific locations in *C. elegans* worms. Specifically, microinjection of a desired DNA construct into the distal gonad is the most widely used method to generate germ-line transformation of *C. elegans*. Although, current *C. elegans* microinjection method is effective to produce transgenic worms, it requires expensive multi degree of freedom (DOF) micromanipulator, careful injection alignment procedure and skilled operator, all of which make it slow and not suitable for scaling to high throughput. A few microfabricated microinjectors have been developed recently to address these issues. However, none of them are capable of immobilizing a freely mobile animal such as *C. elegans* worm using a passive immobilization mechanism. Here, a microfluidic microinjector was developed to passively immobilize a freely mobile animal such as *C. elegans* and simultaneously perform microinjection by using a simple and fast mechanism for needle actuation. The entire process of the microinjection takes ~30 s which includes 10 s for worm loading and aligning, 5 s needle penetration, 5 s reagent injection and 5 s worm unloading. The device is suitable for high-throughput and can be potentially used for creating transgenic *C. elegans*.

## 1. Introduction

*Caenorhabditis elegans* worm is a well-developed model organism for neurodegenerative diseases such as Alzheimer and Parkinson’s [1,2] due to its small size (∼1000 somatic cells), well-mapped neuronal connectivity, transparency, short life cycle (∼2.5 days), and the ability to generate many progeny in a relatively short time. Microinjection is the most reliable method to deliver chemicals, biomolecules and toxins to specific locations inside the target cell or organs in order to probe or perturb the biochemical networks inside the organisms. In case of *C. elegans* worm, microinjection is used to deliver biological reagents such as transgenic constructs and other chemical compounds, into the distal arm of the gonad for applications such as toxicological [3], transgenic [4], drug screening [5] and genetic studies. Current *C. elegans* microinjection methods operate in free space, requires expensive multiple degree of freedom (DOF) manipulators, detailed injector alignment procedures and skilled operator, which makes the injection process slow (10’s of min/worm) and not suitable for scaling to high-throughput. High-throughput microinjection allows larger number of chemical, genetic or pharmacological candidates to be rapidly tested. Through this process, active compounds, antibodies or genes, which modulate a particular bio-molecular pathway, can be identified quickly. For example, high-throughput microinjection can speed up the assays that requires the delivery of the transgenic constructs into the worm, which generally creates mutations in the worm’s offspring.

Recently, microfluidic technology have been used to automate handling of cells, embryos and small organisms as these are of the same size as the microfluidic channels [6,7,8,9,10,11,12,13,14,15,16,17,18,19,20]. More specifically, microfluidic devices have also been used to increase the speed of injection process [21,22,23,24,25,26,27,28,29]. For example, Zhao et. al. [27] demonstrated a microfluidic device with an open chamber for on-chip microinjection of *C. elegans*. In their design, worms were immobilized long-term on the side wall of an open chamber by suction. This can replace the need of halocarbon oil, which is used in conventional microinjection to halt the locomotion of the worm during microinjection. Then an external micro-manipulator was used to perform the microinjection. Although the microfluidic device allowed rapid immobilization of the worms, the system still required active suction to position the worms for injection and precise axial positioning of the gonad was difficult to achieve. Alternatively, Gilleland et. al. [28] presented a computer-assisted microinjection platform, which immobilized the worms in a temperature-sensitive hydrogel using a standard multiwell platform. Then, microinjections were performed under control of an automated microscope using precision robotics. This system could operate at an injection rate of ~25 s/worm with minimal user fatigue. However, the process still needs complex robotic hardware to manipulate the needle-worm in multi-DOF space for obtaining this rate of injection.

In summary, the existing systems either require active immobilization mechanisms that use complex controls or still use multi DOF manipulators to perform accurate injection. An active immobilization mechanisms such as suction [28] or dynamic temperature gradient [29] adds more complexity to the microinjection process while the use of halocarbon oil may not be well suited for high throughput injection and processing of the worms. Therefore, a microinjection device, which can simultaneously immobilize the worm in a passive manner and inject reagents via a simple injection mechanism that is suitable for high throughput microinjection, is required. To achieve this aim, a simple in-plane design that allows visualization of the injection process and increases the speed of the microinjection process was designed, fabricated and tested.

## 2. Device Design

**Figure 1:**
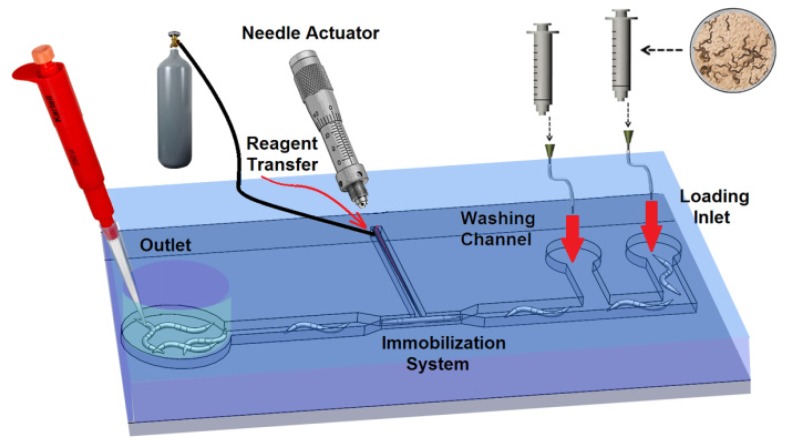
Schematic of the five parts of the microinjector including a loading channel, immobilization mechanism, needle actuation mechanism, reagent delivery system and unloading channel.

The design of the microfluidic microinjector is composed of six parts as shown in Figure 1: (i) Loading channel to transfer the worms from the inlet into the chip, (ii) immobilization channel to stop the movement and locomotion of the worm for injection, (iii) microneedle to create a passage through the worms’ body, (iv) needle actuation mechanism to precisely insert the microneedle into the gonad, (v) reagent delivery system to transfer the desired reagent into worm after needle insertion and (vi) unloading channel (washing channel) to transfer the injected worms from the immobilization channel to the outlet reservoir. To perform this process, first, the young adult *C. elegans* worms were transferred from the agar plate to the device via the loading system. A passive immobilization mechanism (narrowed channel) was used to trap and immobilize the mobile worm for needle insertion. Once the worm was immobilized, the injection needle was precisely moved into the worm for delivery of the reagents by using a single degree of freedom (DOF) compliant mechanism coupled to a micropositioner. Then, the reagent was delivered into the gonad of the worm by means of a capillary pressure microinjection (CPM) technique, which uses pressure driven flow. Finally, the worms were transported to the outlet chamber using M9 buffer from washing channel, and then they were transferred on a feeding agar plate using a micropipette.

### 2.1. Loading System

A loading channel was designed to transfer the young adult *C. elegans* worms (with the length and diameter of 45 µm and 1000 µm, respectively and in-plane sinusoidal swimming pattern with the amplitude of 100 µm) from their feeding culture plate on which they were grown to the injection zone. To do this, a loading system consisting of a syringe, flexible plastic tube and the microchannels on the microfluidic chip was designed (Figure 1). Initially, the worms were washed and transferred from the agar plate by using M9 buffer (3.0 g KH_2_PO_4_, 6.0 g Na_2_HPO_4_, 0.5 g NaCl, 1.0 g NH_4_Cl Bring to 1 L with H_2_O) into a syringe, which was attached to the inlet of the device. Next, the worms were introduced into the device by pressurizing the syringe. The width and depth of loading channel was defined to be 300 µm and 65 µm, respectively. The reservoir diameter was set as 3 mm and the inner and outer diameter of the flexible tube (ID of 1/32” and OD of 3/32”) were chosen. To transfer the worms to the tube, a 3 mL syringe was selected which was controlled by a syringe pump. 

### 2.2. Immobilization Channel

**Figure 2:**
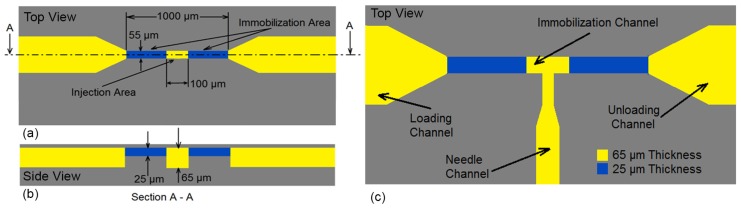
(**a**,**b**) The schematic design of the immobilization system. The narrowed channel had 25 μm depth with 55 μm width. The depth in the middle of the narrowed channel with length of 100 μm was increased to 65 μm where called as “injection area”. (**c**) The microinjector channels composed of four channels: Loading channel, needle channel, immobilization channel and unloading channel.

An immobilization mechanism was required to trap and immobilize the mobile young adult *C. elegans* worm for needle insertion, after loading the worm into the device. To meet this need, an immobilization channel consisting of two narrowed portions at the beginning and end (the blue channels in Figure 2a,b), and one enlarged region at the middle of the immobilization channel (the yellow channels between two narrowed channels in Figure 2a,b), was developed. The two narrowed channel has a depth and width of 25 µm and 55 µm, respectively. Meanwhile, the depth in the enlarged region of the immobilization channel, called as “injection area”, had a length of 100 µm and a depth of 65 µm. The worm was loaded into the immobilization channel by application of a positive pressure at the inlet using the syringe pump. Furthermore, due to compression loading of the worm in the channel, the friction between the worm and the channel walls allow precise micrometer scale position of the worm axially along the channel so that the gonad of the worm can be aligned to the needle.

### 2.3. Needle Size

The function of needle tip is to create a passage through the tissue in which it is inserted. The shape of the tip and its size (inner (ID) and outer diameter (OD) at the tip) play significant roles in tissue-needle interaction and tissue damage. Needles with tip sizes in the range of 3 µm to 6 µm were considered suitable to minimize tissue damage while being large enough to allow easy delivery of reagents into cylindrical shape *C. elegans* worm with diameter of ~45 µm. Unlike conventional *C. elegans* microinjection which uses capillaries with OD/ID of 1000/500 µm for needle fabrication, fused silica microcapillaries with OD/ID of 90/20 µm were chosen for needle fabrication. This selection allows an optimal fit and simple integration of the needle with microfluidic devices where the channel dimensions are 50–100 µm, which was not possible with the needle used in conventional *C. elegans* microinjection. To pull fused silica microcapillaries, a custom-built fused silica capillary pulling machine was designed and built which allows to fabricate conical microneedle with tip sizes in the range of 3 µm to 6 µm.

### 2.4. Needle Actuation

**Figure 3:**
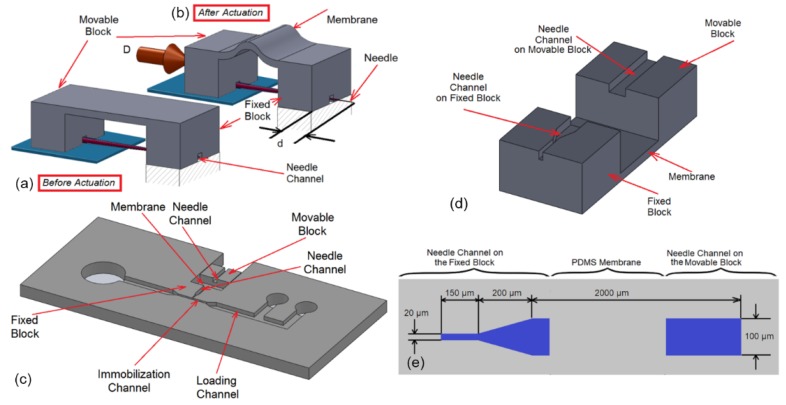
(**a**,**b**) The conceptual design of the compliant mechanism. (**a**) Before actuation. (**b**) After actuation. The motion of the micromanipulator “D” deflected the PDMS membrane subsequently moved “d” the needle inside the needle channel in fixed block. (**c**) This schematic shows how compliant mechanism was integrated to loading and immobilization mechanism. The fixed block was extended and loading and immobilization channel were created on it. (**d**) Bottom view of the complaint mechanism. (**e**) The geometry of the needle channel. The thickness of the channel was uniformly 65 µm.

The goal of the microinjection is to inject biomolecules into the gonad of the worm, when it is immobilized. The gonad of *C. elegans* worm is cylindrical in shape with the diameter and length of 20 µm and 200 µm, respectively. A simple single DOF complaint mechanism (as shown in Figure 3a,b) was used in the place of conventional multiDOF micromanipulator to inject into the distal arm of the gonad. The system was composed of three parts: a movable block, a fixed block and a thin flexible membrane that connects the two blocks (see Figure 3a,b). The microneedle was attached to the movable block and it could move relative to the fixed block inside the needle channel in a manner similar to prismatic joints. When the movable block was pushed by a micropositioner (the displacement “D” in Figure 3b), it deflects the flexible membrane and subsequently, moves the microneedle inside the needle channel (the displacement “d” in Figure 3b). After unloading the movable block, the stored potential energy in membrane (PDMS membrane was used as a spring) drives back the movable block to the stationary point (similar to the condition in Figure 3a).

In order to integrate the complaint mechanism with loading and immobilization system, the fixed block was extended and the loading and immobilization channel were integrated on it as shown in Figure 3c. This design allowed the needle to be well inserted into immobilization channel while its tip was centered with immobilization channel. Since the outer diameter of the shank of the needle was 90 µm, it gently tapers down to the needle tip of 3 to 6 µm, the dimension of the needle channel on the fixed and the movable block was selected as shown in Figure 3e (The depth of the channels was 65 µm). This ensured that the microneedle could smoothly move in fixed block at the tip while being tightly attached to the movable block. 

### 2.5. Reagent Chamber

**Figure 4:**
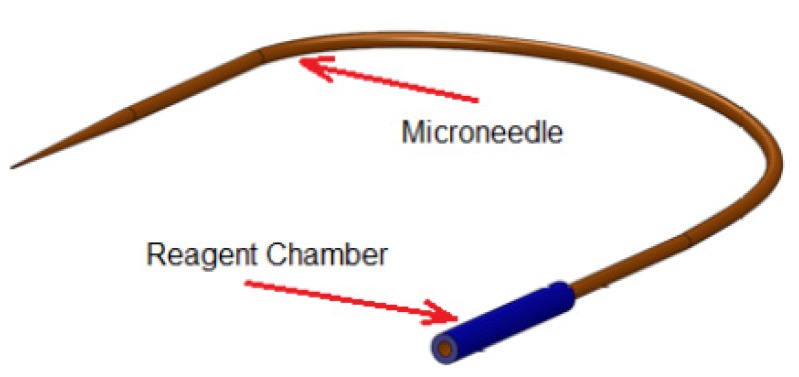
Schematic of the microneedle (OD = 90 μm, ID = 20 μm, length = 80 mm) connected to the reagent chamber (ID = 0.5 (mm), OD = 1 (mm), length = 25 (mm)).

The design of the reagent delivery system is shown in Figure 4. It consists of a reagent chamber and microneedle. In conventional microinjection, the microneedle itself was used as reagent reservoir since the holding volume of the pulled microneedle (typically 50 mm in length and ID of 0.5 mm) was ~9 μL which is larger than the total volume of the reagent required for a set of microinjection (usually ~2 μL). However, the holding volume of the needle (with OD = 90 μm, ID = 20 μm, length of 80 mm) used in this design is extremely small (~25 nL) and could not accommodate all the reagent needed for a set of injections. Therefore, the microneedle was connected to a larger glass capillary (ID = 0.5 mm, OD = 1 mm, length = 25 mm) in order to store the reagent during microinjection as schematically shown in Figure 4. The reservoir can be connected subsequently to either to a pressure source for pressure driven flow. 

### 2.6. Worm Unloading and Plating

Once the reagent was delivered, the worm should be unloaded from the immobilization channel and plated on standard agar plate for recovery. Towards this task, the outlet reservoir was left open to atmosphere by punching a hole such that there are no dead zones for accumulation of the worms as shown previously in Figure 1. When an individual worm was injected, the loading channel was closed and the worm was manually pushed out by using a syringe connected to the washing channel to the open outlet reservoir. The open reservoir also allowed easy pick up of individual worms immediately after injection by using a micropipette and then plate it on agar plate as shown in Figure 1. Then, the injection cycle was repeated to another worm.

## 3. Device Fabrication

**Figure 5:**
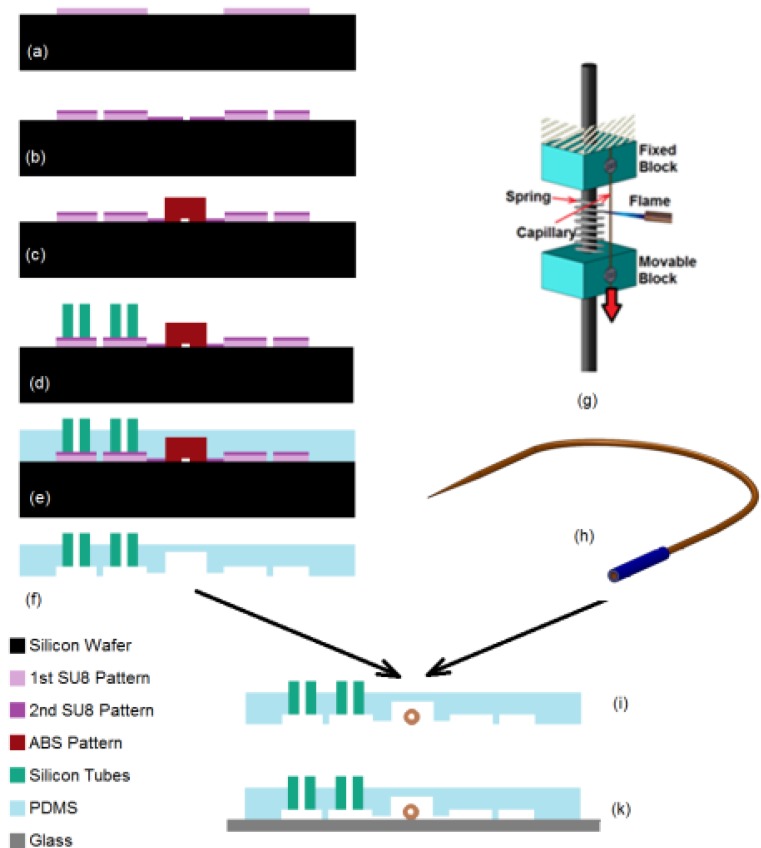
The process flow for device fabrication. (**a**) Pattering of the first SU8 layer with thickness of 40 µm. (**b**) Pattering of the second SU8 layer with thickness of 25 µm on the first layer. (**c**) Attachment of the ABS part (thickness of 2 mm) created by 3D printer to fabricate a hybrid master mold. (**d**) Placement of the interconnectors (silicone tubes) on SU8 pattern. (**e**) Casting of PDMS (1:10) on the mater mold to create a 3 mm device layer and 1mm PDMS membrane for compliant mechanism. (**f**) Peeling off of the PDMS substrate from the master mold. (**g**) Pulling of the microneedle from fused silica capillary and (**h**) Connection of the microneedle to larger capillary to store the reagent. (**i**) Assembly of the microneedle and PDMS chip. (**k**) Bonding of the PDMS chip to the glass slide using dry oxygen bonding.

The fabrication of microinjector device is a multi-step process consisting of PDMS chip fabrication using soft-lithography technique, fabrication of the compliant mechanism and final assembly of the microneedle with the PDMS chip. The process flow for the device fabrication is shown in Figure 5. First, the master mold was fabricated using combination of photolithography process and 3D printer (Figure 5a–c). Next, interconnectors were placed on the master mold and PDMS prepolymer was cast on it (Figure 5d,e). Subsequently, a microneedle was created using a custom-made needle puller and prepared for reagent loading (Figure 5g,h). Then, the PDMS elastomeric chip was peeled of the mold and it was assembled with microneedle. Finally, the chip was bonded to a glass slide (Figure 5f–k).

**Figure 6:**
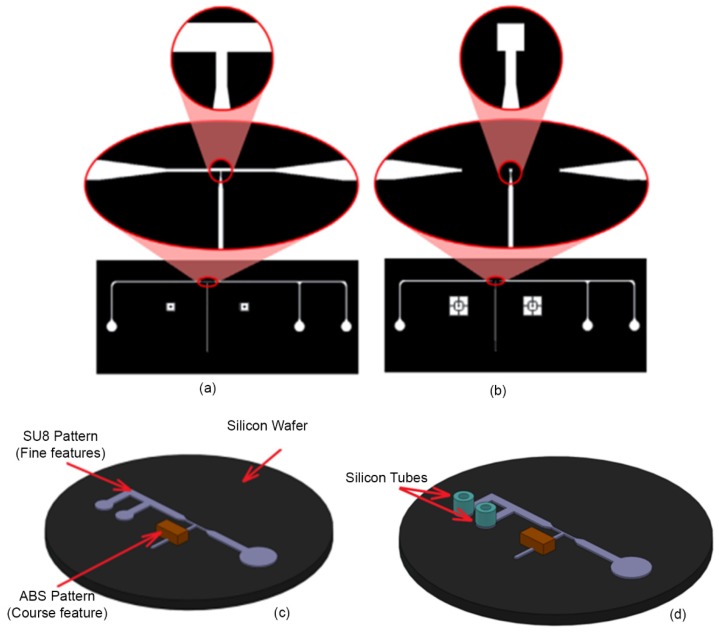
The microchannel pattern for (**a**) the first and (**b**) the second layer of the microchannels. (**c**) The master mold composed of two types of molds (course and fine features). The SU8 pattern on Silicon wafer defined the fine features with resolution of 10 µm and the ABS pattern used to create the course features with minimum resolution of 100 µm. (**d**) The master mold after putting silicon tubes (ID 1.5 mm of and OD of 4.8 mm and 10 mm length) on the SU8 patterns in order to have access to the inlet and washing channel after PDMS casting.

To begin the process, the first layer of the photomask (Figure 6a) was patterned on a 40 µm SU8 (SU-8-2075, Microchem Corp, Newton, MA, USA) that was spun on a silicon wafer (76 mm diameter, University Wafer, South Boston, MA. USA). The same process was repeated to create the second layer of the pattern (Figure 6b) over the first layer with a thickness of 25 µm. In order to create the features related to compliant mechanism, an ABS block with the dimension of 3.5 × 3 mm^2^ base and 2 mm height was 3D printed and attached to the SU8 structures on silicon wafer (at a distance of 1cm from the immobilization channel) to create the final composite mold as shown in Figure 6c. A thin layer (~50 µm) of 1:2 (PDMS to curing agent) PDMS pre polymer was used as the glue. Finally, the mold was heated (75 °C for 1h) to cure the PDMS. In order to have access to the inlet and washing channel, silicone tubes with ID 1.5 mm and OD of 4.8 mm and 10 mm length were used. The tubes were placed on the corresponding reservoirs on the SU8 mold (see in Figure 6c,d). This method allowed complete integration of the interconnects with PDMS device with excellent sealing.

**Figure 7:**
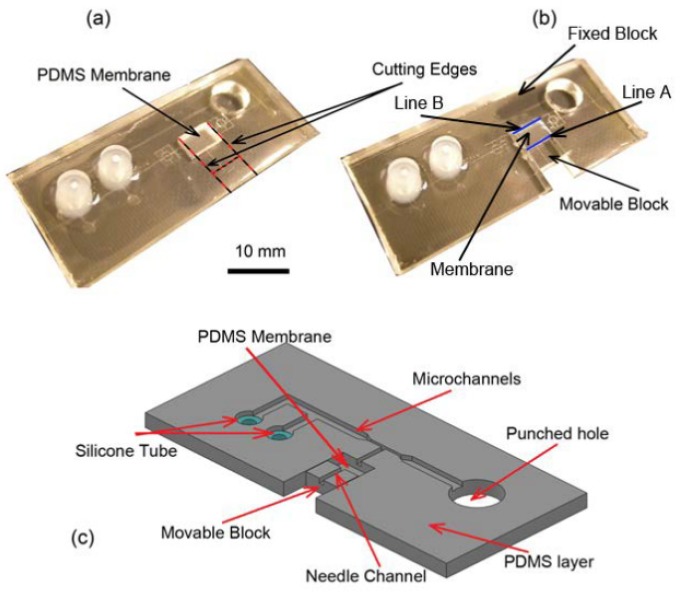
Steps and schematic of PDMS chip cutting and pouching (**a**) The PDMS chip after trimming and pouching. (**b**) The PDMS chip after cutting and releasing the movable block. (**c**) Schematic of different parts of the PDMS chip after casting.

The next step after mold fabrication was PDMS casting. To do this, Polydimethylsiloxane (PDMS) pre-polymer mixture (Sylgard 184 kit, Dow Corning Corp., Midland, MI, USA; 10: 1 ratio of the base and crosslinker) was cast on the master mold and cured at room temperature for 24 h. The volume of the dispended PDMS pre polymer (25 mL) into 10 cm (diameter) Petri dish corresponded to an approximate thickness of 3 mm and 1 mm in the PDMS chip and compliant mechanism section, respectively. Next, the PDMS elastomer was peeled off from the master mold, the extra PDMS was trimmed and the outlet chambers was punched out with a biopsy tool with diameter of 8 mm. Then, the PDMS substrate was cut along the red lines as shown in Figure 7a to make the compliant mechanism. In this configuration, the movable and fixed blocks were connected to the PDMS membrane via “Line A” and “Line B” (blue lines), respectively as shown in Figure 7b. Usually, a small amount of the PDMS prepolymer seeps into the silicone tube during PDMS casting process due to its low surface energy. Therefore, after curing and peeling off the PDMS, the insides of the interconnectors were punched using a biopsy tool to open them. 

**Figure 8:**
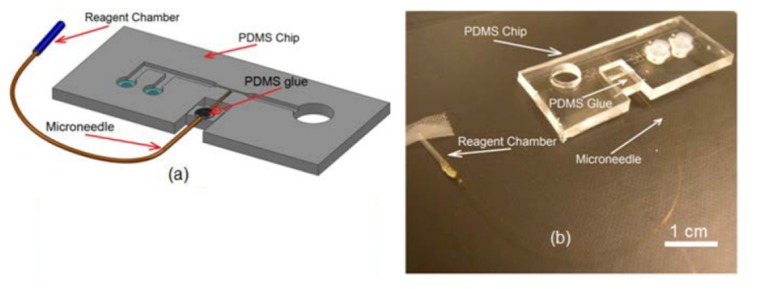
(**a**) A schematic of the needle assembly processes and different parts of the chip. (**b**) The PDMS chip after needle assembly.

The next step is the integration of the microneedle into the PDMS substrate. To do this, the PDMS substrate was inverted and placed under optical microscope (10–20x Objective) such that the microchannel features were accessible from the top for needle integration. Next, the microneedle was gently placed on the PDMS substrate, positioned and aligned so that the tip of the microneedle was at a distance of ~100 µm to 200 µm from the immobilization channel. Then, the shank of the microneedle assembly was inserted into needle channel on the movable block as schematically shown in Figure 8a. Since the size of the needle channel on the movable block was a good fit with the outer diameter of the needle itself, it held the needle tightly at that location and prevented any further movement of the needle during subsequent processes. Next, a droplet (~1 µL) of the PDMS pre polymer (4:1 ratio of the base and crosslinker) was spread on the movable block to fully attach the microneedle to the movable block. After rapid curing the PDMS glue (see the black dot on the movable block in Figure 8a) using a flame, the PDMS chip was taken off from the microscope for bonding (Figure 8b). 

**Figure 9:**
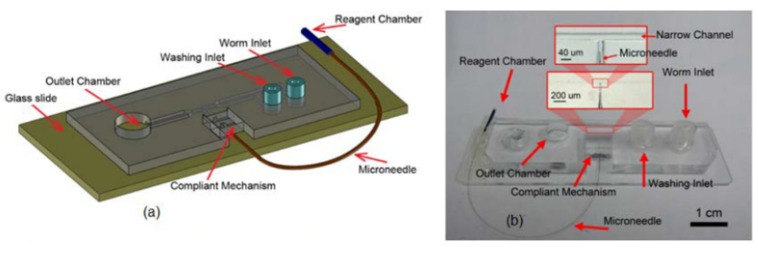
(**a**) A schematic and (**b**) PDMS chip of final *C. elegans* microinjector.

Bonding was the final step in the fabrication of the injection device. The PDMS substrate and a 75 × 25 mm^2^ glass slide were exposed to 50 W oxygen plasma for 70 s. Since the plasma machine worked in low level of the pressure, the DI water present inside the reagent chamber when opening the tip evaporated. Therefore, after taking off the PDMS chip from the plasma machine, the reagent chamber was filled with reagent that is required for microinjection. A thin layer of grease was then spread on the movable block to reduce the friction of the motion during the needle actuation. Afterwards, the PDMS chip was placed under microscope and a 10 mL syringe was connected to the reagent chamber. By pressurizing the reagent chamber, the needle was tested for suitable operation and clogging. If the needle was clogged, a sharp and clean scalpel was used to gentle touch the tip of the microneedle which dislodges any residue accumulated at the tip and opens the microneedle. It is important to be note that the force during the touching should not be high that it breaks the needle. Finally, the glass slide was placed on the PDMS chip and the bonding process was completed (see Figure 9).

## 4. Experimental Setup

**Figure 10:**
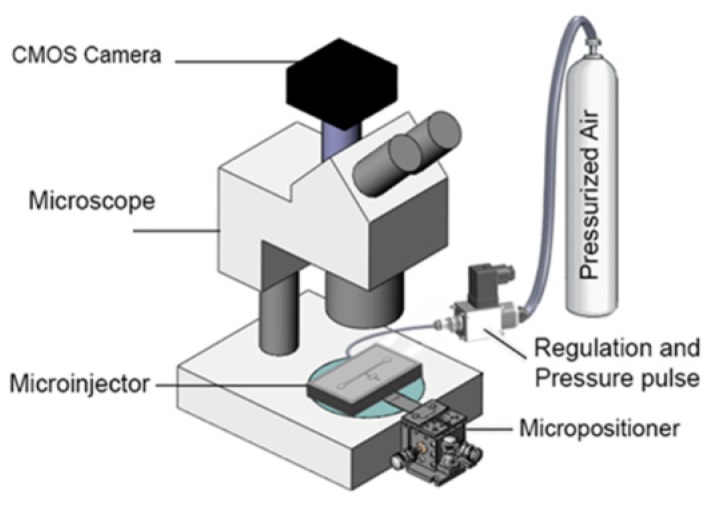
Experimental Setup used for *C. elegans* microinjection consisted of three major parts: Fluidic system, optical system and microchip.

The experimental setup (Figure 10) consisted of three major parts: Fluidic system, optical system and the microfluidic device. The fluidic system which was used to introduce the worms into the device and deliver reagents into the worm, consisted of a pressurized air tank at 4 (bar), pressure regulator (2000 Series Regulator, ARO, Ingersoll Rand, Bryan, OH, USA) and a solenoid valve (S10MM-30-12-3, 3-Way Normally Closed, Pneumadyne, Inc., Plymouth, MN, USA). The optical system that was used to observe, control and record the injection process consisted of an optical microscope (Model 500 LumaScope, Etaluma, Inc., Carlsbad, CA, USA), digital camera (Flea3 FLs-U3-32S2C, FLIR^®^ Systems, Inc., Pittsburgh, PA, USA) and software (Labview^©^, flyCapture2^©^ software, Version 2.13.3.61, FLIR^®^ Systems, Inc., Pittsburgh, PA, USA). The microfluidic device, which was used to perform the injection into *C. elegans* worm, consisted of a microinjector chip (whose fabrication was described in previous chapter) and a micropositioner (Micrometer fine focus linear stage, Model A LHFF, Line Tool Co., Allentown, PA, USA).

The micropositioner attached to the movable block on microfluidic chip (see Figure 9b) was used to accurately move the microneedle with resolution of 5 µm. The air pressure tank was connected to the reagent chamber on microinjector chip through a pressure regulator and a solenoid valve and. This system allows one to generate an adjustable pressure pulse for reagent delivery. The solenoid valve was used to control the duration and number of the pulses for each individual injection. The microfluidic injector was installed under optical microscope (Light Microscope, Leica, Concord, ON, Canada) and a CMOS camera mounted on it was used to record the video of the injection processes. After injection, a 200 µL micropipette was used to transfer the injected worms into the standard nematode growth (NG) agar plates for recover after injection. 

## 5. Results and Discussion

### 5.1. Characterization of the Immobilization System

**Figure 11:**
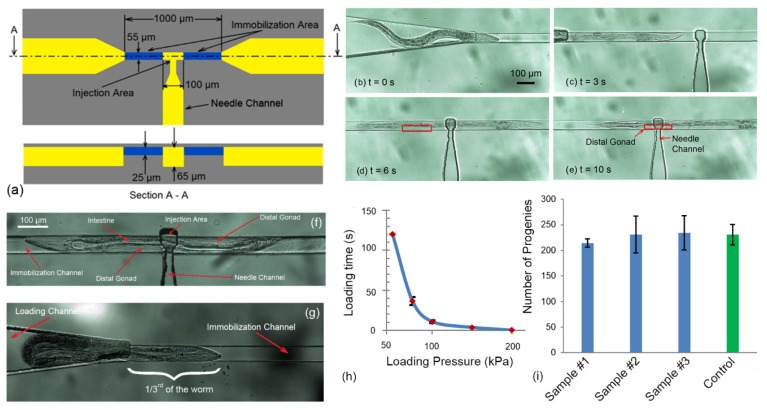
(**a**) A schematic of the immobilization channel. The narrowed channel had 25 µm depth with 55 µm width. The depth in the middle of the narrowed channel with length of 100 µm was increased to 65 µm where called as “injection area”. (**b**–**e**) image sequence of worm loading process. (**b**) The worm was introduced into the immobilization channel, (**c**) the worm was pushed into the immobilization channel, (**d**) the worm was fully inserted into the immobilization channel and (**e**) the distal gonad of the worm was aligned with needle channel for needle insertion. (**f**) an immobilized *C. elegans* worm inside the final design. The picture shows that the narrowed portions of the immobilization channel would allow easy visualization of the internal organs as well as allow consistent immobilization. (**g**) At the loading pressure less than 50 (kPa), the worms could not be fully inserted into immobilization channel. When 50 (kPa) pressure was applied on the worms, only 1/3rd of the length of the worm could be compressed into narrowed channel and the rest of worm was remained in loading channel. (**h**) The loading time versus the loading pressure. At *t* = 120 s, the worm was not fully loaded and at *t* = 0 s, the worm was not captured in the immobilization channel. (**i**) Viability of the worms after immobilization. Worms (n = 5 for each plate) reproduction rate 72 h after 5 min immobilization compared to not immobilized control animals.

To immobilize the worm, first, the wild-type N2 *C. elegans* worms was moved into the mouth of the narrowing region of the immobilization channel via loading channel (Figure 11b) by using a constant pressure on the syringe. Then, the pressure was applied to push the worm into the immobilization channel (Figure 11c). The worm was compressed on the sides via a two narrowed channel with the depth and width of 25 µm and 55 µm, respectively. The enlarged region of the immobilization channel, called as “injection area”, had a length of 100 µm and a depth of 65 µm (see Figure 11a). Subsequently, the position of the worm in the channel and its alignment with the position of the needle channel was adjusted by inserting (Figure 11d) or withdrawing (Figure 11e) the plunger in the syringe as appropriate. The narrowed portions of the immobilization channel would allow easy visualization of the internal organs as well as allow consistent immobilization; while the enlarged region at the center which is connected to the needle channel, will allow centered injection as shown in Figure 11f.

For a certain defined immobilization channel (25 µm depth and 55 µm width here), the required time to introduce and align the worms (relative to the needle) into the immobilization channel was dependent on the applied pressure. In order to characterize the required pressure with loading time, constant pressure in range of 50–200 kPa was applied inside the loading channel to load the worm in the immobilization channel. Subsequently, the immobilization process (Figure 11b–e) was observed and recorded from which the required loading time was calculated. The result of the experiment, loading time versus the loading pressure, has been plotted in Figure 11h. The results showed that at 50 kPa loading pressure or below, the worms could not be fully inserted into immobilization channel. When 50 kPa pressure was applied on the worms, only 1/3rd of the length of the worm could be compressed into narrowed channel and the rest of worm was remained in the loading channel as shown in Figure 11g. The experiment was performed on 5 worms and after 120 s, the worms could not be inserted more than 1/3rd of its length. 

In the immobilization process, there are two forces acting on the worm. One is the force due to pressure that pushes the worm through the narrow channel. The other is the frictional force on the worm’s body. The fiction force is dependent to the length of the worm, inserted into the channel. At low pressures (<50 kPa), the frictional force dominates over the applied pressure. Therefore, the worm stops to move at the certain length (~1/3rd of the worm here) as shown in Figure 11g. At the other extreme, at 200 kPa loading pressure or higher, the worm could not be trapped by narrowed channel and all worms (n = 5) passed through the narrow channel (the loading time was reported as zero for these situation). The reason was that the loading pressure significantly exceeded the frictional resistance and the worm was pushed past the immobilization channel. The pressure ranges from 75–150 kPa allowed a relatively fast loading process with reasonable immobilization control inside the narrowed channel for all worms (n = 5) in this geometry. At this pressure range, the balance between pushing and friction forces was well controllable. Since this immobilization mechanism was of a passive design, the friction force caused by immobilization channel on certain *C. elegans* worm was dependent to the geometry of the channel. Therefore, the optimal pressure range for immobilization can be changed to the lower pressure range by increasing the width and/or depth of the immobilization channel and vice versa. The required time to load the worms varied from 37 s to 3.5 s when the pressure was increased from 75 kPa to 150 kPa.

Although, compression was effective to immobilize the worms inside the narrowed channel, it could potentially have an impact on its viability. In order to study the effect of mechanical stress caused by compression into narrowed channel (in this specific geometry) on the viability and reproduction rate of the worms, a total of 15 young adult Wild-type N2 *C. elegans* worms were loaded into narrowed channel consecutively and immobilized. Then they were kept inside the channel for 5 min and subsequently unloaded and distributed evenly (5 worms) on three agar plates (standard nematode growth (NG) agar plates containing OP50 *Escherichia coli* as a food source) to study the viability and reproduction of the worm after 72 h. A similar number of worms from the same batch was cultivated on the agar plates without putting them through the microdevice and used as control for comparison. The number of progenies on the plate after 72 h was counted for each plate. The results demonstrate that the worms recovered well after being immobilized transferred to *E. coli*-bacteria (OP50) seeded agar plates (100% viable after 72 h). As compared to control sample, they moved normally and were indistinguishable from the control animals in their movement behavior (the frequency of their curling movement was visually compared with control sample and no significant difference was observed). Moreover, their reproduction (shown in Figure 11i) was not significantly affected in compared with control sample (*p*-value = 0.3058) which indicated that there was minimal stress or tissue damage due to immobilization in this format.

### 5.2. Characterization of Compliant Mechanism

**Figure 12:**
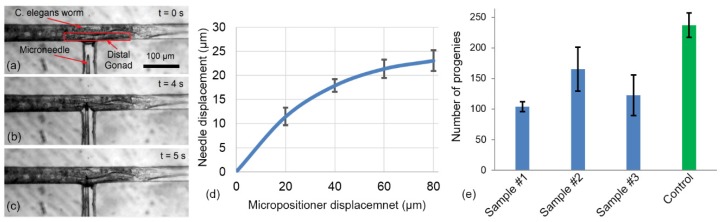
(**a**–**c**) image sequence of the needle insertion, (**a**) the microneedle was aligned with distal gonad of the worm, (**b**) the microneedle was moved and penetrated into the worm, (**c**) the microneedle was inserted into the gonad and suitable for the reagent delivery. (**d**) The characterization of the compliant mechanism. A known displacement has been applied to the micropositioner and measuring the movement of the microneedle was calculated using images of the tip of the microneedle. The experiment has been repeated for 8 times on one device. The minimum resolution of the micropositioner was 20 µm. (**e**) Viability test of the worms after needle insertion. Worms (n = 5 for each plate) reproduction rate 72 h after needle insertion compared to not inserted control worms.

The compliant mechanism provides a simple yet quick method for insertion of needle into the worm, In this mechanism, the needle tip is moved from its original position into the immobilization channel in fewer than 5 s (Figure 12a–c). The performance of the compliant mechanism attached to the micropositioner was characterized by applying a known displacement to the micropositioner and measuring the actual movement of the microneedle tip. The needle motion was recorded under microscope and then, the videos were analyzed to obtain needle displacement by using a custom image processing procedure. The plot in Figure 12d shows the displacement applied by the micropositioner (x-axis) and the resultant needle motion as measured through image analysis (y-axis). Since the initial position of the needle was not the same for all the devices, the needle position was normalized to the original position. It can be seen that the microneedle can be moved in a controllable and repeatable manner by using a micropositioner and a compliant mechanism. The relationship between the displacement of the needle tip and that of the micropositioner is not linear. This may be because the stiffness of the micropositioner was not constant and varied with the displacement. Similar to the conventional microinjection, the depth of the injection can be controlled by operator in this device. An insertion depth of about 5–25 µm from the body wall of the worm is generally aimed for to achieve a successful injection. A marker for successful injection into the gonad would be the physical expansion of the gonad when the pressure pulse is applied. In the cases where such an expansion is manifested the delivered reagents have remained in the gonad. An unsuccessful injection leads to spread of the reagents into other body parts, notably the gut. Therefore, according the characteristics shown in in Figure 12d, the compliant system designed in this platform is capable to perform accurate needle insertion into the gonad.

In order to study the effect of the combination of immobilization and injection on the viability and reproduction rate of the worms, a set of experiment was performed, as described below. Young adult Wild-type N2 *C. elegans* worms (n = 15) were immobilized into narrowed channel and then the microneedle was inserted into the gonad for 2 s and then removed. Subsequently, the worms were unloaded and plated to examine the effect of the microneedle insertion and the ensuing tissue damage on the viability and reproduction.

The results (see Figure 12c) demonstrated that number of the progenies for the injected worms were significantly (*p*-value < 0.00001) reduced (105–165 progenies) compared to the control worms (238 progenies) due to the effect of immobilization and injection (the error bar in Figure 12c shows the standard deviation from three sample that were taken to count the number of the progenies). Since the immobilization alone does not cause any significant difference in the number of progenies, it can be concluded that the needle insertion and the ensuing tissue damage cause the reduction in the number of progenies produced by the worms. This decrease in the number of progenies was similar to the case of other microinjection procedures for *C. elegans* where the progeny size is reduced to 10–50% of the normal brood size. Observation of the worms post injection on agar plate showed that the worms were able to recover normal motion and were indistinguishable from the wild type within 24 h after needle insertion.

### 5.3. Characterization of Reagent Delivery 

**Figure 13:**
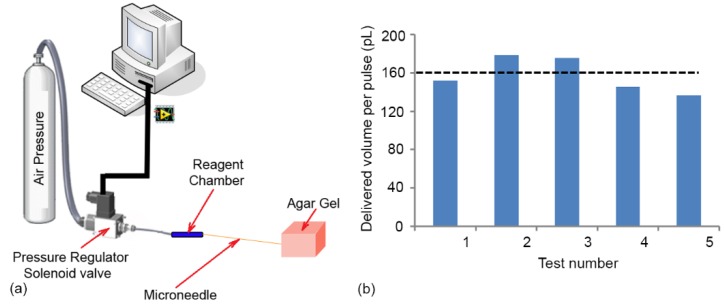
(**a**) Schematic of the experiential setup used to characterize the reagent delivery system. The microneedle (OD = 90 µm, ID = 20 µm, length = 80 mm) connected to the reagent chamber (ID = 0.5 (mm), OD = 1 (mm), length = 25 (mm)). (**b**) The characterization of the reagent delivery system. For each pulse 200 kPa was applied for a duration of 1 s. In average, 160 pL reagent can be delivered for each pulses. By controlling the duration of the pulses or the pressure level, the delivered volume can be controlled to achieve lower injected reagent per pulses.

In pulse pressure driven flow method, the volume of reagent delivered can be controlled by changing the pressure level as well as the duration of the pulse. In order to characterize the delivery characteristics of the system, the microneedle was inserted into a block of agar gel (1%) to mimic the consistency of the interior of the worm (see Figure 13b). The duration of the pressure pulse was set to 1s and the pressure regulator connected to the gas cylinder was used to control the pressure level (200 kPa). Methylene blue dissolved in DI water (0.01 M) was used as a model injection reagent to visualize the movement of the liquid. Since the volume delivered in each pulse was small and cannot be easily visualized, a set of 1000 pulses (frequency of 0.5 Hz) were delivered and the total volume injected calculated by the displacement of the solution interface in the reagent chamber. The volume delivered in each pulse was calculated by simply dividing the total volume delivered by the number of pulses applied and is plotted for five different attempts in Figure 13b. The results show that the delivery system was consistently able to deliver ~160 pL per pulse and the variation between different attempts was small which meets the design criteria for delivery. A typical worm at its prime reproductive stage has a volume of ~1.4 nL. In conventional microinjection, injected volumes were ~15% (200 ± 20 pL) or more of the worm’s body volume. Therefore, the volume of the regent chamber which was ~1.5 µm would be sufficient for ~100 worm injection with a reasonable dead volume of the system

### 5.4. Microinjection of the Worm and Its Visualization

**Figure 14:**
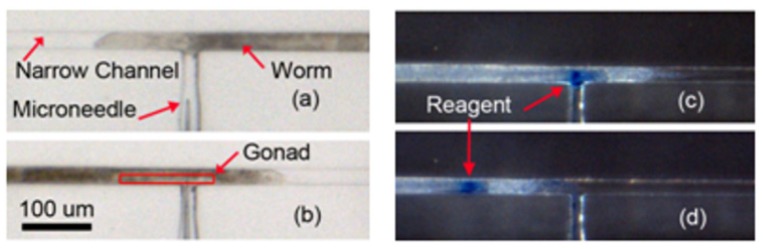
The sequence of four steps for microinjection: (**a**) loading and immobilization, (**b**) needle penetration, (**c**) reagent injection and (**d**) unloading.

In order to visualize the delivery of reagents into the body of the worm, Methylene blue (0.05 M) was injected into the distal arm of the gonad using the microinjection device. The sequence of steps involved in the microinjection process is shown in Figure 14. The L4 wild-type N2 *C. elegans* at 56 h were loaded at the inlet. Subsequently, they were pushed into the immobilization channel and one of the two gonads was aligned with the tip of the needle (Figure 14a), pneumatically under visual feedback. Following this, a micropositioner connected to the complaint mechanism was used to gradually move the needle and penetrate the distal end of the gonad (Figure 14b). Then, by applying 2 pressure pulses with the magnitude of 200 kPa and 1 s duration, the reagent in the microneedle was delivered into the gonad (Figure 14c). Afterwards, the worm was unloaded and transferred into outlet chamber (Figure 14d).

The results showed that the reagent could be successfully injected into the worm and the dye was distributed in worm after injection. As illustrated in Figure 14d, the injected dye did not come out of the injection spot during the unloading process, which indicated that compression pressure in the immobilization channel did not affect the ability of the dye to remain in the worm in a significant way. However, visualization with Methylene blue did not provide sufficient contrast to identify the movement of the dye inside the worm post injection. Therefore, a DNA solution bound to fluorescent dye was used in the next section to study the distribution of the dye into the gonad after injection. 

### 5.5. DNA Injection

**Figure 15:**
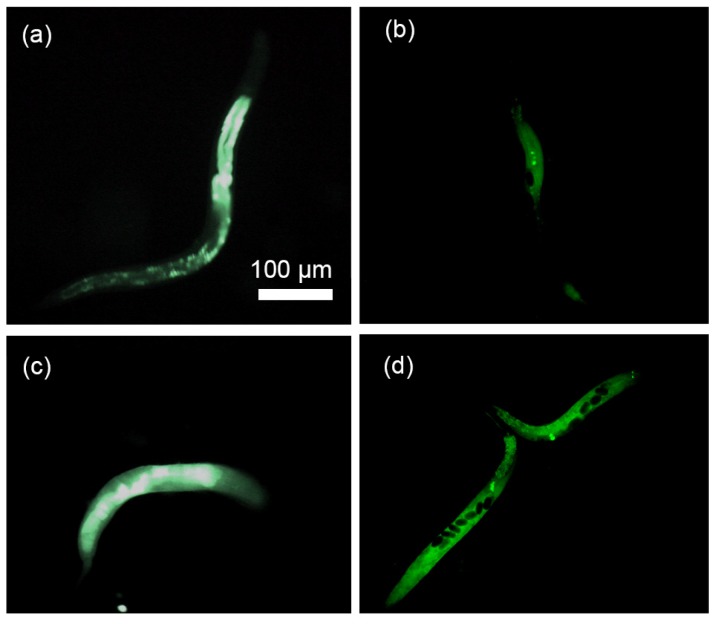
Fluorescent DNA solution was delivered into gonad (**a**,**b**) and the intestine (**c**,**d**) in both conventional (**right**) and microfluidic method (**left**). The scale bar shown in (**a**) is the same on (**b**–**d**).

A DNA solution tagged with green fluorescent dye was injected into the gonad using the same process as described in previous section in order to visualize the distribution of the dye inside the body of the worm post injection. First, the fluorescent DNA reagent consisted of 1 µL UltraPure™ Salmon Sperm DNA Solution (concentration of 10 mg/mL, Thermofisher scientific, Brampton, ON, Canada), 1 µL Acridine Orange (concentration of 10 mg/mL, SIGMA-ALDRICH, Oakville, ON, Canada) and 1 mL DI water was prepared (the components were mixed and the solution was kept 30 min at room temperature and then stored into −20° fridge). Acridine Orange is a nucleic acid binding dye that emits green fluorescence when bound to dsDNA such as sperm (red fluorescence when bound to ssDNA or RNA). Next, the 2 µL reagent chamber was filled with the sample solution. Then, the Wild-type N2 *C. elegans* were loaded, injected and unloaded as described previously and the fluorescent image of the injected worms was recorded and shown in Figure 15a,c. In order to compare, other worms were injected at multiple locations using conventional method and the fluorescent image of the injected worms were recorded and shown in Figure 15b,d.

**Figure 16:**
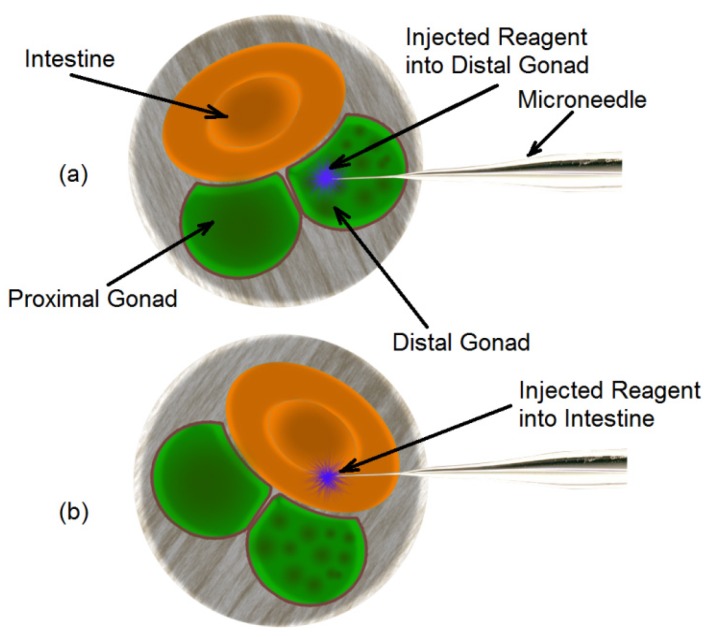
The cross-section of the young adults *C. elegans* and its alignment relative to the microneedle inside the microinjector. (**a**) The microneedle is properly aligned with the distal gonad (**b**) the microneedle is aligned with the intestine instead of distal gonad and the reagent is delivered into the intestine.

It can be seen from Figure 15a,b that the DNA solution was located to a small region in the body, while in the case of Figure 15c,d, the reagent was distributed throughout the body of the worm. This can be explained by the location of delivery. Figure 15a,b are typical when the injection occurred in the gonad which is located in the mid-section of the worm. Figure 15c,d are typical of delivery into the intestine which extends throughout the entire stretch of the worm. In order to identify the probability of injection into the gonad, a group of 30 young adult *C. elegans* worms were immobilized, gonad aligned to the microneedle and injected with DNA solution in the microdevice. Analysis of fluorescent images post injection revealed that 63% (19 worms) of the injections were delivered into the gonad and 37% (11 worms) into the intestine. The success rate in this system was higher than the performance of conventional microinjection (success rate of 30%) [30]. The reason that 37% of the injections into the intestine can be explained based on the cross sectional orientation of the worm body with the microneedle tip at the time of injection as shown in Figure 16. The cross-section of young adult *C. elegans* worm shows that if the orientation of the gonad were aligned with needle (Figure 16a), the injection would happen into the gonad. However, any misalignment between the needle and the gonad would lead the needle injection into the intestine of the worm (see Figure 16b) which was happened into 37% of the injections in this experiment. These two situations cannot be distinguished from each other, as the visualization mechanism is top down and 2D.

## 6. Conclusions

In summary, a microfluidic device for microinjection of *C. elegans* worm was developed. This microinjector was capable of simultaneously passive immobilizing a freely mobile animal such as *C. elegans* and performing microinjection by using a simple and fast mechanism for needle actuation. The entire process of the microinjection takes ~30 s which includes 10 s for worm loading and aligning, 5 s needle penetration, 5 s reagent injection and 5 s worm unloading. Compared to conventional microinjection which takes tens of minutes to perform one injection, this system can increase the speed of the microinjection significantly. The integration of the 1 DOF complaint mechanism with the passive immobilization channel, could significantly simplify microinjection process compared to conventional methods. The delivery system could precisely deliver 160 pL of the reagents into the gonad. The results showed that the DNA solution could be successfully (with success rate of 63%) injected into the distal gonad. However, the DNA solution used in this experiment was only a model reagent to track the distribution of the reagent after injection. Similar injection but with plasmids can be used to create transgenic worms.
